# Perioperative Management of a Patient With Left Ventricular Free Wall Rupture After Myocardial Infarction: A Rare Case Scenario

**DOI:** 10.7759/cureus.29368

**Published:** 2022-09-20

**Authors:** Gurpreet Panesar, Vishal V Bhende, Tanishq S Sharma, Sunil K Karna, Manish Tiwari, Kunal A Soni, Kartik B Dhami, Nirja Patel, Hardil P Majmudar, Sohilkhan R Pathan

**Affiliations:** 1 Cardiac Anesthesiology, Bhanubhai and Madhuben Patel Cardiac Centre, Bhaikaka University, Karamsad, IND; 2 Pediatric Cardiac Surgery, Bhanubhai and Madhuben Patel Cardiac Centre, Bhaikaka University, Karamsad, IND; 3 Adult Cardiology, Bhanubhai and Madhuben Patel Cardiac Centre, Bhaikaka University, Karamsad, IND; 4 Cardiac Surgery, Bhanubhai and Madhuben Patel Cardiac Centre, Bhaikaka University, Karamsad, IND; 5 Clinical Research Services (CRS), Bhanubhai and Madhuben Patel Cardiac Centre, Bhaikaka University, Karamsad, IND

**Keywords:** cardiac rupture, intra-aortic balloon pump, pericardial effusion, cardiac tamponade, mechanical complications post-myocardial infarction, left ventricular free wall rupture

## Abstract

Myocardial infarction (MI) is typically followed by numerous lethal complications. One such complication is left ventricular free wall rupture (LVFWR). We present the case of a middle-aged hypertensive patient who had a history of unstable angina for seven days. He presented to the emergency room with chest pain, dyspnea, and unstable vital parameters. Clinical signs, electrocardiography, and echocardiography raised the suspicion of left ventricular free wall rupture with ST-segment elevation inferior wall and lateral wall MI. As a result, the patient received aggressive resuscitative measures. Later, he underwent surgical repair for cardiac rupture via cardiopulmonary bypass. Finally, the patient was discharged from the hospital on the 10^th^ postoperative day. The window period from the onset of cardiac wall rupture to patient admission to the operating room is crucial. This case report highlights that a high index of suspicion for left ventricle free wall rupture should be considered for a patient presenting with MI and cardiogenic shock. Timely diagnosis and quick surgical intervention can deter complications and save the patient.

## Introduction

Left ventricular free wall rupture (LVFWR) is a rare but catastrophic sequel, complicating 2%-4% of acute myocardial infarctions (MIs) [1]. The incidence of cardiac rupture has decreased to 0.5%-2% due to the widespread and successful use of primary percutaneous coronary interventions [2]. These mechanical complications usually occur within 24 h to seven days after acute MI but could occur as late as one-month post-myocardial infarction [3].

LVFWR should be suspected in patients with recent ischemic events who present with severe persistent angina, hemodynamic instability, vomiting, and syncopal attacks (due to arrhythmia or transient electromechanical dissociation).

Herein, we present a case of cardiac rupture in a middle-aged man who had mechanical complications after the first episode of MI, a proven risk factor for LVFWR.

## Case presentation

Approval for this study was given by the Institutional Ethics Committee (IEC-2), HM Patel Centre for Medical Care and Education, Anand, Gujarat Approval No. IEC/BU/2022/Cr.07/89/2022 dated 28/05/2022. Additionally, written informed consent for publication was obtained from the patient.

We present a case of a 55-year-old male with hypertension, no known addictions, and past history of a cerebrovascular accident resulting in left-sided hemiparesis three years ago. It was an ischemic infarct that recovered completely with conservative management. Recently, he had seven days of recurrent chest pain, which was treated conservatively by a local physician. However, he presented to our emergency room in a drowsy but arousable condition with severe chest pain, acute onset dyspnea of 30 minutes, and diaphoresis. His vital signs were non-recordable pulse and blood pressure (BP), tachypnea, unrecordable saturations by a pulse oximeter, and cold peripheries.

As immediate resuscitative measures, we secured wide bore intravenous access to administer fluid boluses at 10-20 ml/kg/hr, up to 3 liters until shifting the patient to the operation room. Inj. noradrenaline was started at 0.1 mcg/kg/min intravenously. The patient’s respiration was supported with non-invasive ventilation (NIV) with 100% oxygen. An electrocardiogram (ECG) revealed sinus tachycardia, ST elevation in I, II, III, aVF, V4-V6, and ST depression (upsloping) in V5-V6 suggestive of acute ST elevation inferior wall and lateral wall MI (STEIW/LWMI). Aspirin 300 mg (tablet), clopidogrel 300 mg (tablet), and atorvastatin 80 mg (tablet) were given through Ryle’s tube. Then, the patient was transferred to the cardiac center with NIV and inotropic supports.

**Figure 1:**
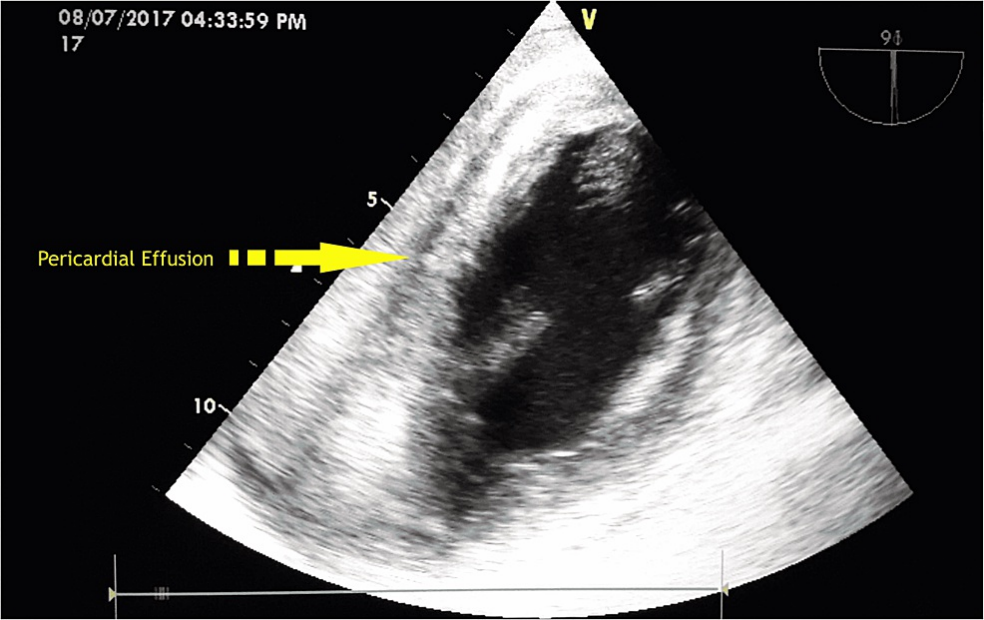
Echocardiographic image showing pericardial effusion

Echocardiography showed severe hypokinesia in the inferoposterolateral ventricular wall. It also revealed a large heteroechogenic collection in the pericardial cavity with organized echogenic mass compressing the right ventricular cavity (suggestive of an organized clot) (Figure 1).

**Figure 2:**
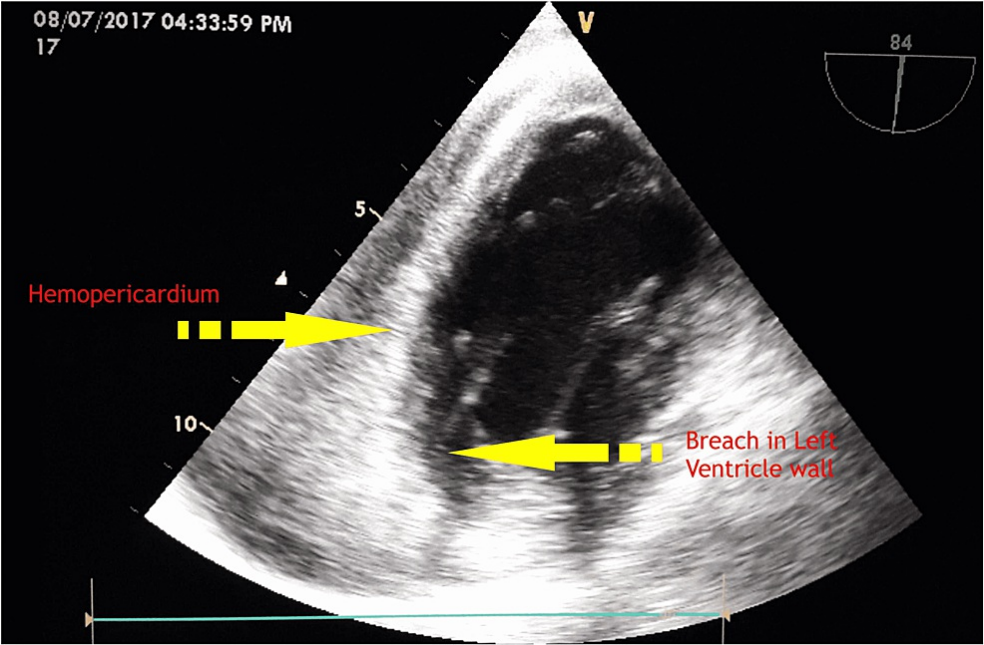
Echocardiographic image showing a breach in the continuity of the inferolateral myocardial wall

In addition, there was a breach in cardiac wall continuity at the inferolateral wall with some Doppler flow, raising the possibility of cardiac wall rupture. However, the left ventricular ejection fraction was 30% (Figure 2).

The diagnosis of acute LVFWR as a mechanical complication of MI was made based on the patient’s hemodynamics status, signs of pericardial tamponade, ECG suggestive of ST elevation inferior and posterior wall myocardial infarction, and echocardiographic suspicion of LVFWR. The patient’s relatives were counseled for a poor prognosis, and the cardiothoracic surgeon was informed about the operation. The consent for the American Society of Anaesthesiology risk score of 5 (ASA 5) was obtained, and the patient was transferred to the operating room for emergency surgery.

In the operation room (OR), the patient was anesthetized with etomidate 6 mg IV, fentanyl 50 mcg IV, midazolam 0.5 mg IV, and rocuronium 100 mg IV. The patient was intubated, and bilateral air entry was confirmed. We inserted a triple lumen in the right internal jugular vein, followed by the insertion of a transesophageal echocardiography probe. Cardiopulmonary bypass (CPB) was instituted, and blood cardioplegia was given after applying an aortic cross-clamp. Details of the sequence of events and the cardiopulmonary bypass from the time the patient reported until his discharge have been mentioned in Tables 1, 2.

**Table 1:** Timeline of events since patient admission (Time mentioned as per Indian Standard Time) SICU: Surgical Intensive Care Unit; CPB: Cardiopulmonary Bypass;

Events	Time
Patient reporting to coronary care unit (Cardiac Centre)	14.30, Postoperative - Day 0
Shifted to OR	15.45, Postoperative - Day 0
Institution of CPB	16.51, Postoperative - Day 0
Shifting to SICU	20.00, Postoperative - Day 0
Extubation time	12.15, Postoperative - Day 1
Intermittent mandatory ventilation time	16 hours
Intensive care unit stay	3 days
Hospital stay	10 days

**Table 2:** Cardiopulmonary bypass (CPB) details

Pump Time	1 hour 51 minutes (111 minutes)
Cross-clamp time	1 hour, 21 minutes (81 minutes)
Prime volume	1500 ml.
Urine on CPB	3000 ml.
Conventional ultrafiltration / Modified ultrafiltration	2200 ml.
Blood products	2 packed cell volume
Fluid balance	850 ml. negative balance
Cardioplegia	St. Thomas cardioplegia solution

**Figure 3:**
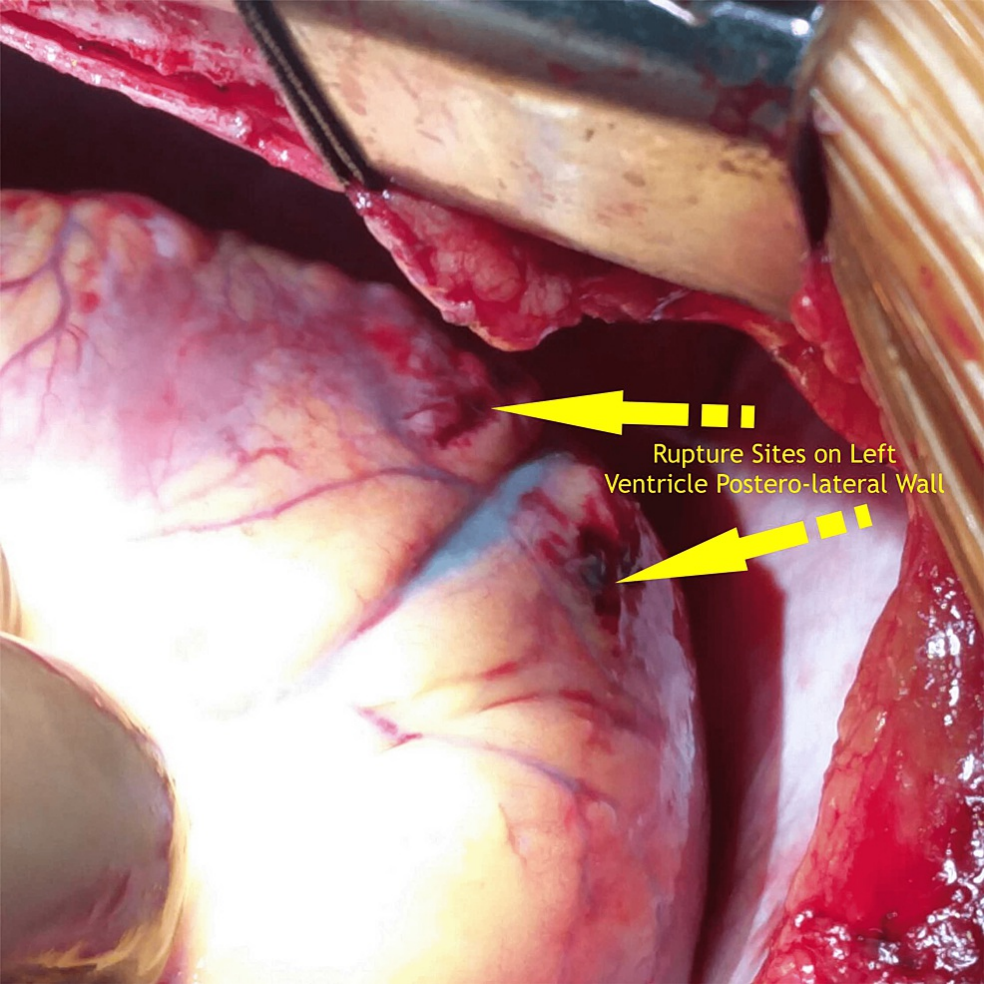
Intraoperative image showing two sites of left ventricular perforation

**Figure 4:**
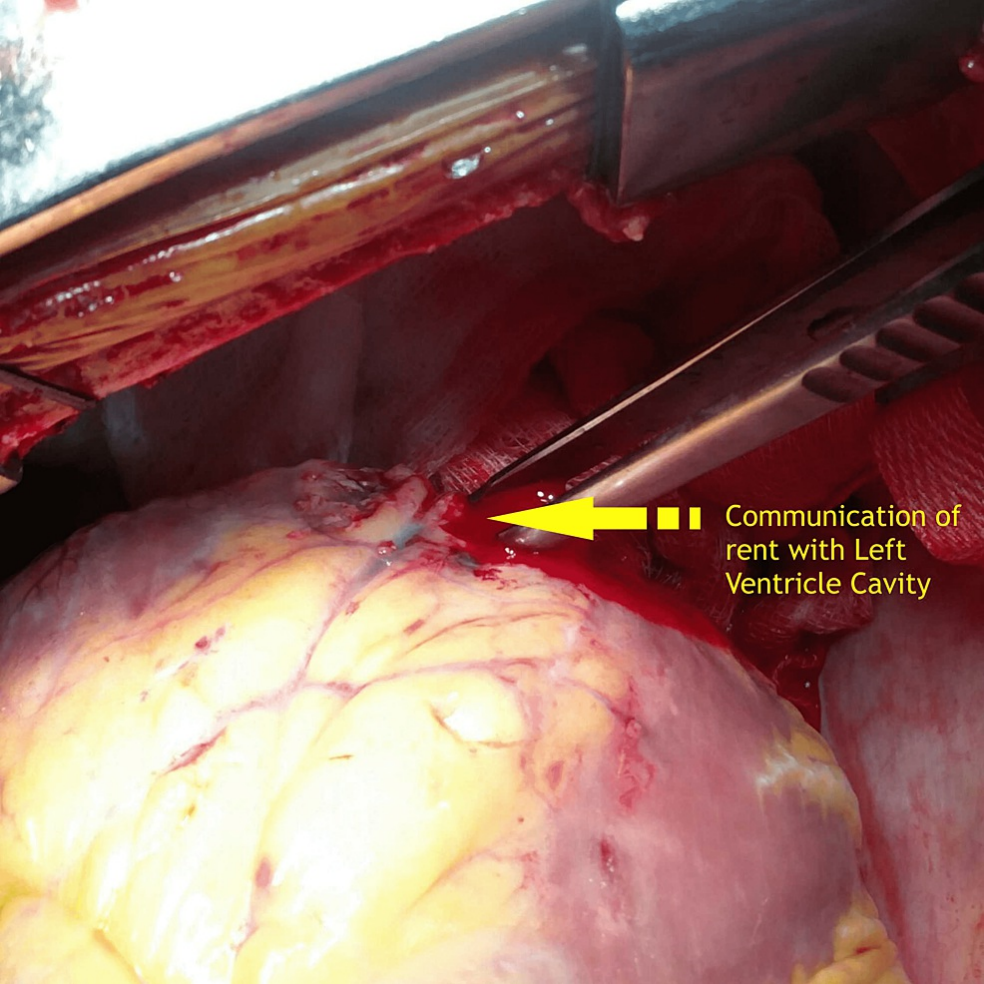
Intraoperative image showing communication of the perforation with the left ventricular cavity

Intraoperative findings revealed two sites of rupture in the left ventricular posterolateral wall, supplied by an obtuse marginal, with the rent communicating with the left ventricular cavity (Figures 3, 4).

**Figure 5:**
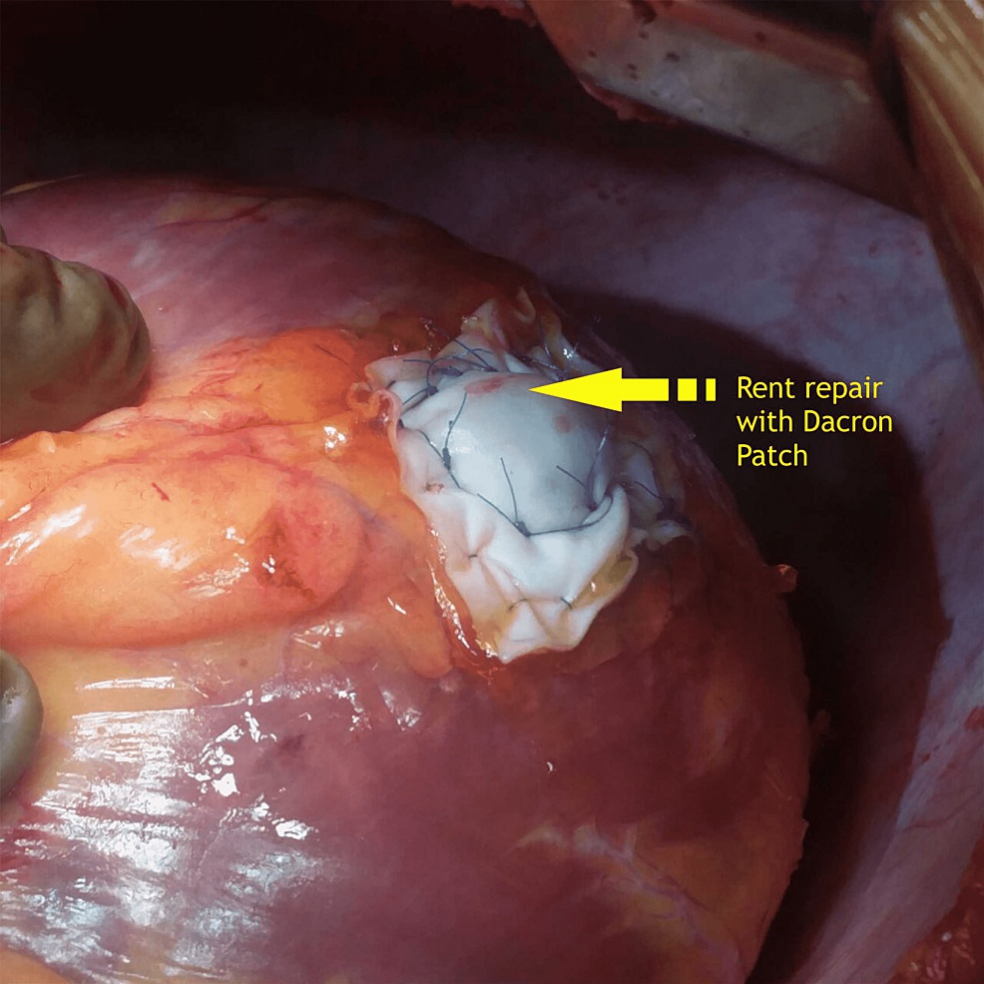
Intraoperative image showing repair of left ventricular perforation done with double patch technique

The dead myocardium surrounding the LV rupture was partially excised, and both the rents were merged into one large rent. The rent was repaired using the “double patch technique” with a circular Dacron patch inside the left ventricular cavity and a bovine pericardium patch on the LV surface, with the rent and infarcted myocardium sandwiched between the two patches sutured in place using a prolene 5/0 suture (Figure 5). Bioglue was applied liberally to cover the patch and surrounding area.

**Figure 6:**
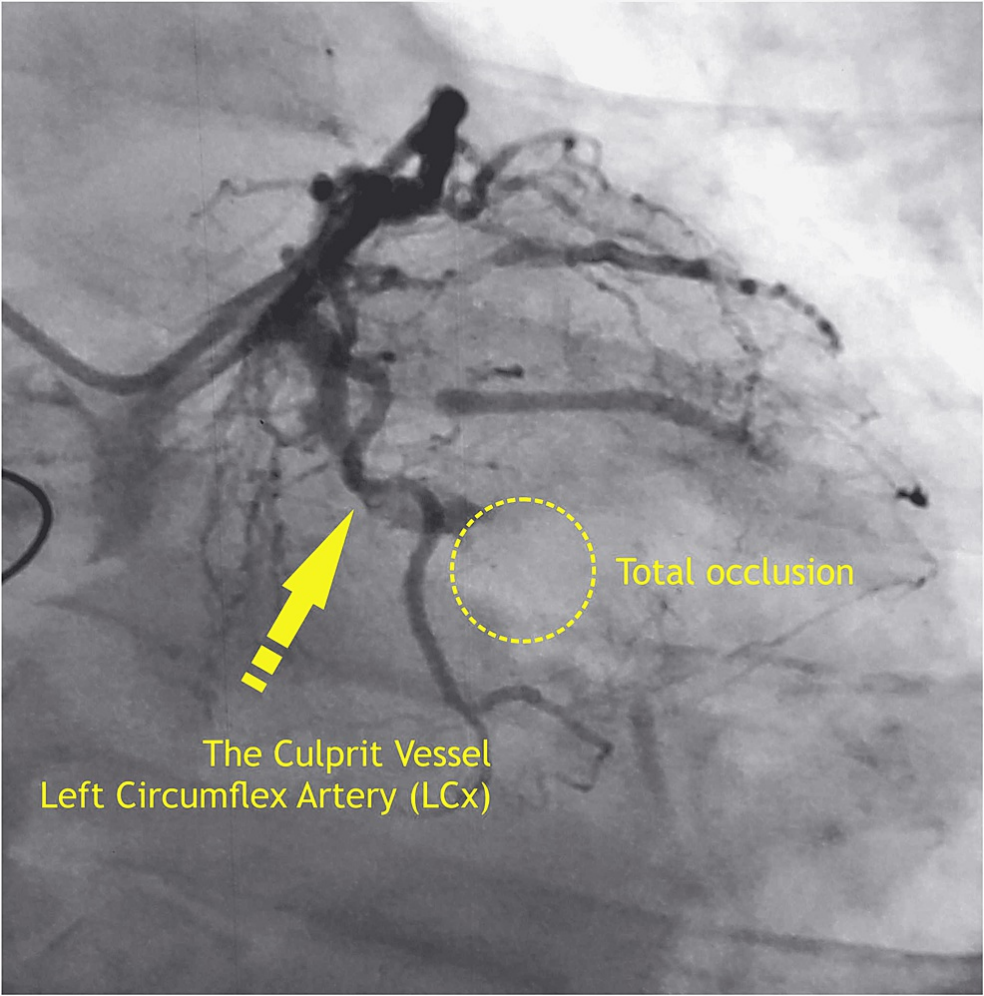
Angiography showing complete occlusion of the culprit vessel (left circumflex artery)

The aortic cross-clamp was released and prophylactic inotropes - inj. dobutamine 5 mcg/kg/min and inj. noradrenaline 0.1 mcg/kg/min - were started. The patient was weaned from CPB uneventfully and received one packed cell volume, two fresh frozen plasma, and two cryoprecipitates after weaning off from CPB in the operating room. Later, the patient was transferred to the ICU on moderate inotropic support. He was extubated on the first postoperative day and inotropes were tapered off slowly. Coronary angiography on the third postoperative day revealed double vessel disease in the left circumflex and right coronary arteries (Figure 6).

As the left circumflex was the culprit’s vessel, it was decided not to intervene immediately. However, the patient was discharged on the 10th postoperative day with a hemodynamically stable condition and showed a 30% LV ejection fraction on echocardiography

After 45 days, the patient was readmitted, and percutaneous transluminal coronary angioplasty with stenting to the right coronary artery was successfully performed. When the patient came for a follow-up after three months, he was asymptomatic, and LV function improved from 30% to 40%. Two years later, patient outcomes were promising, with a left ventricular ejection fraction of 45%.

## Discussion

Here, we discuss a case of a 55-year-old hypertensive patient who complained of unstable angina for seven days but didn't get himself properly investigated. He presented to us with severe chest pain, dyspnea, diaphoresis, and unstable vital signs. With the aid of ECG and echocardiography, he was diagnosed with LVFWR with ST elevation inferior wall myocardial infarction (STEIWMI)/ST elevation lateral wall myocardial infarction (STELWMI) and cardiogenic shock. Prompt resuscitative measures were started, and surgical repair of the ruptured site was done. Later, the patient was discharged on the 10th postoperative day.

The incidence of all mechanical complications is higher (0.27%) in ST-segment elevation MI (STEMI) compared to (0.06%) non-ST-segment elevation MI (NSTEMI), primarily due to the extensive transmural necrosis of myocardium [4].

Ventricular septal rupture is the most common mechanical complication post-MI, followed by papillary muscle rupture; free wall rupture is the least common among the three [4]. However, the mortality rates of free wall rupture range from 75% to 90% compared to 40%-75% for ventricular septal rupture, making the management of cardiac rupture more challenging [5].

Common risk factors for LVFWR include advanced age (age > 60 years), gender (female), presence of hypertension without left ventricular hypertrophy, first episode of MI, delayed or inadequate reperfusion, and use of corticosteroids and non-steroidal anti-inflammatory drugs (NSAIDS) during the active phase [6,7]. Early reperfusion, either with thrombolytic therapy or successful percutaneous coronary intervention, limits the infarction’s extent, saves the epicardium from necrosis and helps against rupture. In contrast, NSAIDs and corticosteroids reduce fibroblast proliferation and halt connective tissue regeneration, thus favoring the rupture of the infarcted area [8,9].

Due to the poor prognosis and high peri-operative mortality, free wall myocardial ruptures witnessed even in hospitals are left and no surgical correction is done [10]. Here, we highlight the timely management of an outside hospital subacute cardiac rupture post-MI with cardiogenic shock.

The New Clinical classification for ventricular free wall rupture given by Wei Gong et al. also endorses the salvageability of patients with LVFWR [11].

Acute or cardiac arrest type is the acute form of LVFWR that occurs in 83% of cases and is characterized by sudden circulatory collapse, hypotension, and electro-mechanical dissociation, with imminent death unless resuscitative measures are started promptly. The subacute or unstable type occurs in 9.6% of the patients with myocardial rupture with an 87.5% mortality rate. The ruptured site is sealed either by a hematoma or a layer of the epicardium, leading to pseudoaneurysm formation. As a result, those patients present with pericardial effusion signs and sudden angina, transient arrhythmia, syncope, and transient electromechanical dissociation.

The stable type represents 7.7% of patients with free wall rupture (16.7% mortality rate) who usually present with persistent hypotension or refractory angina with moderate or massive pericardial effusion [11]. Diagnostic pericardiocentesis confirms fluid in the pericardium. This subgroup has a good prognosis with surgical interventions. The free wall rupture can also be classified according to the morphological features as Type-I, where an abrupt slit-like tear generally occurs within 24 h of acute MI [12]. Type-II represents an erosion of infarcted myocardium, suggesting a slow tear of dead myocardium, which occurs 24 h after infarction. Type-III is an early aneurysm formation with subsequent rupture in the late phase of MI.

The complete rupture of the myocardium leads to death due to hemopericardium and cardiac tamponade. In contrast, incomplete rupture may either progress to frank rupture with pericardial tamponade, end up with pseudoaneurysm formation, have communication with the left ventricle, or form a left ventricular diverticulum [13].

To confirm LVFWR in patients, the preferred diagnostic modality is standard bedside transthoracic echocardiography. Apart from pericardial effusion with tamponade (suggested by chamber collapse and dilated inferior vena cava), the actual myocardial tear at the rupture site can also be visualized [12]. If the patient’s condition allows, a computed tomography (CT) scan can help demonstrate the extravasation of contrast into the myocardium, confirm the diagnosis, and locate the rupture site. Left ventriculography performed during cardiac catheterization is a crude diagnostic approach, as it may enhance the ongoing leak and deteriorate the patient’s status [14]. In contrast, cardiac magnetic resonance imaging (MRI) can strengthen the clinical diagnosis, but its feasibility depends on the patient’s clinical condition.

Patients with LVFWR require immediate and aggressive resuscitative measures until they are wheeled into the operating theater for emergency corrective repair. Fluids should be started at 10-20 ml/kg/hr and inotropes are required to maintain good systemic vascular resistance like noradrenaline 0.05-0.2mcg/kg/min.

Inj. dobutamine 5-10 mcg/kg/min or inj. adrenaline 0.05-0.1 mcg/kg/hr should be started intravenously to improve myocardial contractility. Respiration is supported with NIV with fractional inspired oxygen (FiO2), depending on the patient’s saturation. Pericardiocentesis is helpful, as it deters the threat of cardiac tamponade and ensures the patient’s clinical stability before surgery. Preoperative insertion of an intra-aortic balloon pump may help by reducing the afterload of the struggling left ventricle, as mentioned in the guidelines of the European Society of Cardiology (ESC) 2017 [15].

The surgical intervention has high perioperative mortality rates equal to 20%-75%; however, it results in increased survival and good quality of life, as seen in 10 years of follow-up by Wei Gong et al. [11]. On the contrary, the patients treated conservatively reported only a 10% survival rate [16]. The goal of surgery is not only to stop bleeding and relieve tamponade but also to prevent a second rupture.

Infarctectomy is routinely followed, involving extensive debridement up to normal cardiac tissue and then direct linear closure or end ventricular patch plasty, as done in our case [15].

Recently, techniques that avoid the institution of cardiopulmonary bypasses, such as using biocompatible hemostatic glues or patches to cover the infarcted area, have been used with good outcomes [17]. However, after sutureless repair of ischemic myocardium, pseudoaneurysm is a common long-term reported sequela [18].

Revascularization of the infarct artery in patients with STEMI is advocated along with the repair of myocardial rupture, as multivessel coronary artery disease is present in most cases of free wall rupture as recommended in 2021 American College of Cardiology (ACC)/American Heart Association (AHA) guidelines. However, revascularization of non-infarct arteries in a patient with STEMI complicated by shock is not recommended, as it can potentially increase the risk of death and renal failure [19]. So, right coronary artery (RCA) plasty was done after 45 days in our case. The angiography benefit has to be weighed against the risk of clinical deterioration and even death due to treatment delay. If the clinical condition permits, coronary angiography can be performed, but if not feasible, empirical bypass grafting of all main coronaries is also documented in the literature [20]. The current availability of hybrid theaters, which makes intraoperative coronary angiography feasible, is a boon for such patients.

## Conclusions

LVFWR can occur a few hours to days after MI but can also be the presenting complaint of a patient admitted to the emergency room. Keeping a high level of suspicion in mind, bedside echocardiography for MI patients presenting with cardiogenic shock can help diagnose the condition early. Once diagnosed, adopting aggressive resuscitative measures and immediate surgical intervention can give promising outcomes.
